# Intrinsic Photosensitivity Enhances Motility of T Lymphocytes

**DOI:** 10.1038/srep39479

**Published:** 2016-12-20

**Authors:** Thieu X. Phan, Barbara Jaruga, Sandeep C. Pingle, Bidhan C. Bandyopadhyay, Gerard P. Ahern

**Affiliations:** 1 Department of Pharmacology and Physiology, Georgetown University Medical Center, 3900 Reservoir Road, NW, Washington DC, 20007, USA; 2 Department of Biology, Vinh University, Vinh City, Vietnam; 3 Research Service, Veterans Affairs Medical Center, Washington, DC 20422, USA

## Abstract

Sunlight has important biological effects in human skin. Ultraviolet (UV) light striking the epidermis catalyzes the synthesis of Vitamin D and triggers melanin production. Although a causative element in skin cancers, sunlight is also associated with positive health outcomes including reduced incidences of autoimmune diseases and cancers. The mechanisms, however, by which light affects immune function remain unclear. Here we describe direct photon sensing in human and mouse T lymphocytes, a cell-type highly abundant in skin. Blue light irradiation at low doses (<300 mJ cm^−2^) triggers synthesis of hydrogen peroxide (H_2_O_2_) in T cells revealed by the genetically encoded reporter HyPerRed. In turn, H_2_O_2_ activates a Src kinase/phospholipase C-γ1 (PLC-γ1) signaling pathway and Ca^2+^ mobilization. Pharmacologic inhibition or genetic disruption of Lck kinase, PLC-γ1 or the T cell receptor complex inhibits light-evoked Ca^2+^ transients. Notably, both light and H_2_O_2_ enhance T-cell motility in a Lck-dependent manner. Thus, T lymphocytes possess intrinsic photosensitivity and this property may enhance their motility in skin.

Organisms have evolved a multitude of photoreceptors tuned to different light frequencies and coupled to diverse cellular responses. Plants, algae, bacteria and protozoa express red, blue/green and UV-light receptors that mediate photosynthesis, phototropism and phototaxis^1^. In addition to specialized eyes, fish, amphibians and reptiles use photoreceptors in the pineal organ and skin to regulate melatonin synthesis and pigmentation respectively^2^,^3^. Until recently, photoreception in mammals was believed to be restricted to the eye. Only rods and cone cells in the retina (mediating the visual pathway) and a sub-population of retinal ganglion neurons (mediating circadian sensory input and pupillary constriction) were thought to contain true photoreceptors^2^,^3^. Human skin is nonetheless a major target for sunlight. UV light striking the superficial, epidermal layer triggers several well-described phototoxic and photochemical reactions, including the synthesis of Vitamin D and melanin^2^. Interestingly, functional rhodopsin-containing photoreceptors have been identified in human melanocytes^4^ and keratinocytes^5^ and may contribute to UV phototransduction. Less energetic, visible radiation (400–750 nm) penetrates much deeper into the dermis than UV light with an e-fold reduction in intensity only every ~1 mm^6^. Some of this visible irradiation is reflected off blood vessels back through the skin, such that the total radiant flux near the skin surface is greater than the incident light^6^. Thus, compared with UV light, visible light can affect a much greater range of cells. Indeed, recent studies showed that red light stimulates the proliferation of keratinocytes^7^ and fibroblasts^8^ while blue light alters skin cell differentiation^9^.

Sunlight also has a prominent effect on immune cells and immune function leading to beneficial effects on human health, including reduced incidence of autoimmune diseases^10^,^11^,^12^ and cancers^13^. The precise mechanisms by which sunlight affects immune function are unclear. Although, enhanced synthesis of Vitamin D is one possibility^14^,^15^, there is emerging evidence for Vitamin D-independent effects of sunlight^16^,^17^,^18^. Notably, normal skin contains a high density of T lymphocytes (~1 × 10^6^ cells cm^−2^) performing immune surveillance, and the total number of T cells resident in skin is estimated to be double of that in circulation^19^. The effects of light on T cells, however, remain unexplored. In this study we report that T cells possess intrinsic sensitivity to blue and UV light. The detection of light is coupled to generation of H_2_O_2_ and activation of Src kinase and PLC-γ1 leading to elevated intracellular [Ca^2+^]. Photosensitivity is greater in activated T cells and enhances T-cell motility. Thus, T cells are a new type of photoreceptive cell and their photosensitivity may contribute to the effects of sunlight on immune function.

## Results

### Blue light increases [Ca^2+^] in T cells

**Figure 1:**
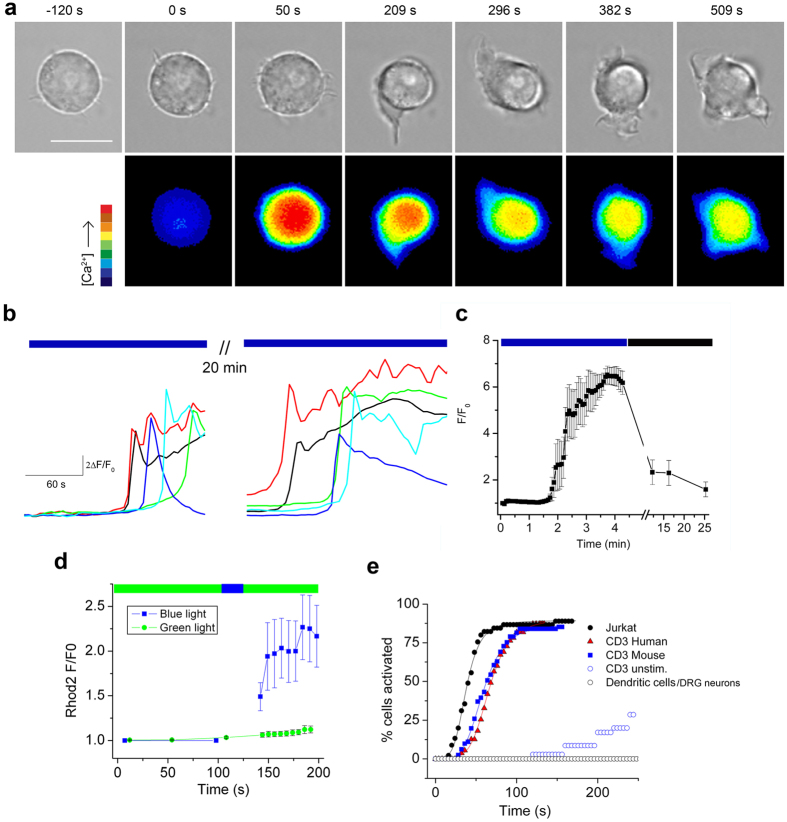
Blue light increases intracellular [Ca^2+^] in T cells. (**a**) Phase (561 nm) and Fluo-4 fluorescence (488 nm, average 2.7 mW cm^−2^, 37 °C) images of a Jurkat cell show light-induced increase in intracellular [Ca^2+^] followed by extension of lamellipodia. Scale bar = 10 μm. (**b**) Representative traces of Fluo-4 fluorescence (F/F_0_) in Jurkat cells during irradiation with 20 mW cm^−2^ blue light (480 ± 15 nm) and after 20 minutes recovery (in dark). (**c**) Mean Fluo-4 fluorescence during blue-light irradiation (20 mW cm^−2^) and recovery in dark; n = 20. (**d**) Brief blue-light stimulation (27 mW cm^−2^, 20 s) increases Ca^2+^ measured by Rhod-2 fluorescence (excitation 540.5 ± 12.5 nm, 3 mW cm^−2^, n = 10 cells) (**e**) Comparison of light sensitivity in T cells (blue light, 17 mW cm^−2^, 31 °C). Plot shows the cumulative% of cells activated (>20% change from baseline) by continuous blue-light irradiation measured from 2 s bins (n = 30–50 cells per group). Smooth lines are best fits to a Hill function used to obtain the time for half-maximal activation (T_1/2_); Jurkat (38 s), Con A-stimulated human CD3+ (59 s), Con A-stimulated mouse CD3+ (66 s) cells, un-stimulated mouse lymph node CD3+ cells, mouse bone-marrow derived dendritic cells and DRG neurons (no response).

We observed that blue-light irradiation of Jurkat T cells triggers an increase in intracellular [Ca^2+^] as measured by Fluo4 fluorescence (Fig. 1a and b, Supplementary Movies 1 and 2). The Ca^2+^ responses decreased after recovery in dark (20 min), after which cells could be re-stimulated by light (Fig. 1b and c). The responses were not due to photosensitization of Fluo4 since a blue-light pulse produced a similar effect when Ca^2+^ was measured using Rhodamine-2 with green light excitation (Fig. 1d). A cumulative activation plot shows that blue light (17 mW cm^−2^) increased [Ca^2+^] in ~90% of Jurkat T cells with a time for half-maximal activation (T_1/2_) of 38 seconds (Fig. 1e). We observed a similar response in human and murine CD3+ T cells that had been previously activated by plate-bound anti-CD3 antibody or Concavalin A treatment (Fig. 1e). In contrast, un-stimulated T cells exhibited a greatly diminished light sensitivity. Further, light had no effect on murine, bone-marrow derived dendritic cells nor cultured sensory DRG neurons (Fig. 1e).

### Irradiance and spectral dependence of T-cell photosignaling

**Figure 2:**
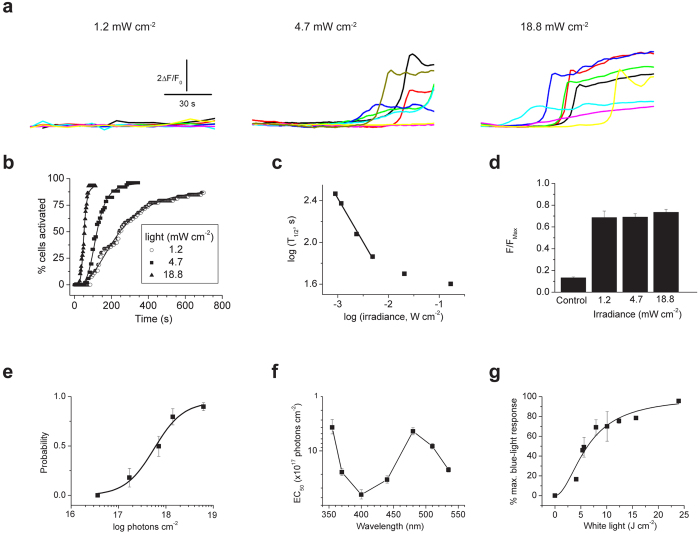
Energy and spectral dependence of T-cell light signaling. (**a**) Fluo-4 fluorescence (F/F_0_) in Jurkat cells during 90 s irradiation with 1.2, 4.7 and 18.8 mW cm^−2^ blue light (480 ± 15 nm). (**b**) Cumulative% of cells activated by continuous blue-light irradiation measured from 2 s bins (n = 30–50 cells per group). Smooth lines are best fits to a Hill function used to obtain the time for half-maximal activation (T_1/2_). (**c**) Plot of log T_1/2_ versus log irradiance. The linear fit shows that T_1/2_ is proportional to irradiance at low energy (**d**) Peak Fluo4 fluorescence (normalized to ionomycin, *F*_Max_) evoked by different irradiances (2–20 minutes). Data are mean of 10–13 cells per group. Control represents *F* at time zero. (**e**) Fraction of cells responding to varying doses of blue-light irradiation (60 s, 31 °C, n = 40–50 cells for each point). Half-maximal activation (EC_50_) is 5.6 ± 1.2 × 10^17^ photons cm^−2^ or 232 mJ cm^−2^. (**f**) Plot of EC_50_ values for Ca^2+^ rise evoked by irradiation of different wavelengths; n = 40–60 cells for each point. (**g**) Changes in Fluo-4 fluorescence evoked by broad-spectrum (400–700 nm) irradiation. Data (30–50 cells) are normalized to maximal response evoked by blue light. Smooth line shows best fit to a Hill equation with an IC_50_ of 6.7 ± 0.6 J cm^−2^.

Figure 2a–c show that the latency for light evoked responses depended on the irradiance. At low intensities (<6.5 mW cm^−2^ or 1.5 × 10^16^ photons s^−1^ cm^−2^) the T_1/2_ was directly proportional to the photon fluence rate (Fig. 2c). At higher irradiances the T_1/2_ approached saturation that may reflect a dead time in the signaling pathway. In contrast, Fig. 2d shows that the peak [Ca^2+^] of ~70% of a maximal ionomycin stimulus, was independent of the irradiance (1.2 to 18.8 mW cm^−2^). This peak response was comparable to, if not greater than, the Ca^2+^ rise evoked during TCR-mediated signaling (Supplementary Fig. 1). These data show that the light response depends on the total number of photons rather than irradiance flux or time and this observation supports the existence of a photon-counting mechanism.

Dose-response analysis for light-induced Ca^2+^ revealed half-maximal blue-light activation with 232 mJ cm^−2^ or 5.6 ± 1.2 × 10^17^ photons cm^−2^ (Fig. 2e). To measure the spectral sensitivity for light signaling we irradiated T cells with light of varying wavelengths between 355 and 535 nm and constructed dose-response relationships for each wavelength as in Fig. 2e. Figure 2f shows a plot of EC_50_ versus wavelength (an action spectrum), and reveals peaks at ~355 nm and ~480 nm, with a sharp falloff at wavelengths greater than 510 nm. Thus, T cells exhibit discrete sensitivity to UV and visible-blue light, and their photosensitivity does not correlate with photon energy or heating. We also measured the T cell sensitivity to full-spectrum white light (400–700 nm). Figure 2g shows that visible radiation activated Ca^2+^ levels with a half-maximal response at 6.7 ± 0.6 J cm^−2^. Importantly, these low doses of blue or full spectrum light were not toxic to T cells; we observed no changes in cell viability and murine splenocytes irradiated (up to 5 J cm^−2^ white light) exhibited normal proliferation to plate bound anti-CD3ε (Supplementary Fig. 2).

### Light generates Ca^2+^ signals via a Src kinase/PLC-γ1 pathway

**Figure 3:**
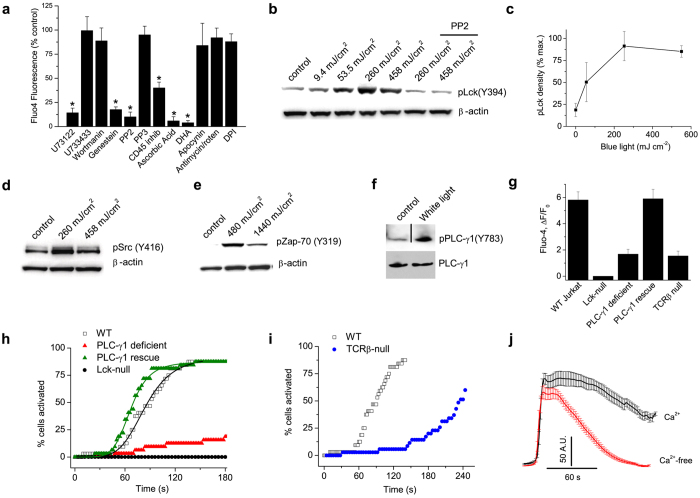
Light activates a Lck-Phospholipase C-γ1 pathway. (**a**) Summary of Ca^2+^ responses evoked by blue light (4.7 mW cm^−2^, 450 mJ cm^−2^) in Jurkat cells pretreated (see Methods) with U73122 (500 nM), U733433 (5 μM), wortmanin (1 μM), genestein (100 μM), PP2 (3 μM), PP3 (3 μM), CD45 inhibitor (1 μM), ascorbic acid (10 mM), DHA (docosahexaenoic acid, 5 μM), apocynin (100 μM), antimycin/rotenone/oligomycin (20: 20:5 μM), DPI (diphenyliodonium, 50 μM) (n = 30–40 cells per group in triplicate), *P < 0.001. (**b** and **c**) Blue light dose-dependently increases phosphorylation of Lck(Y394) in Jurkat cells detected with a generic pSrc (Y416) antibody (see Supplementary Fig. 3), data are mean of 3 experiments. The Src inhibitor, PP2, blocks the increase in pY394 demonstrating that light triggers trans autophosphorylation of Lck. (**d**) Blue light increases pSrc in murine CD3^+^ T cells. (**e**) Blue light triggers phosphorylation of ZAP-70 (Y319) in Jurkat cells. (**f**) White light (13.7 J cm^−2^) stimulates tyrosine phosphorylation (Y783) of PLC-γ1. Note that the immunoblots are cropped and the full blots are shown in Supplementary Fig. 9. (**g–i**) Mean changes in Fluo4 fluorescence and cumulative activation plots in response to blue light for wild-type Jurkat cells, PLC-γ1-deficient Jurkat T cells (Jgamma1), Jgamma1 cells stably expressing PLC-γ1 (JgammaWT), Lck-deficient Jurkat cells (JCam1.6) and cells lacking TCRβ expression (n = 40–120 for each group). (**j**) Light-evoked Ca^2+^ responses in control and Ca^2+^-free media; n = 40–50. The rising phase of responses is aligned to show the time course of decay.

Next, we tested a range of pharmacologic inhibitors to explore how light sensing generated Ca^2+^ transients in T cells. Figure 3a shows that the broad-spectrum tyrosine kinase inhibitor, genestein, the Src tyrosine kinase inhibitor, PP2, a protein phosphatase CD45 inhibitor and the PLC inhibitor, U73122, all blocked light-evoked Ca^2+^-responses in Jurkat cells. During T cell receptor (TCR) signaling the Src kinases Lck and Fyn, phosphorylate several tyrosines in the CD3 zeta chain leading to the recruitment and activation of the protein kinase, ZAP-70^20^. We found that blue-light dose-dependently stimulated phosphorylation of Lck, the dominant Src kinase in Jurkat cells^21^ (Fig. 3b and c, Supplementary Fig. 3). Levels of phospho-Lck increased beginning at 50 mJ cm^−2^ and peaked at 260 mJ cm^−2^ and this paralleled the light-dependent increase in [Ca^2+^] (see Fig. 2e). Induction of phospho-Lck was completely inhibited by PP2 indicating that light causes transphosphorylation of Lck (Fig. 3b). Similarly, blue light activated phospho-Src in primary CD3+ T cells (Fig. 3d). Additionally, blue (Fig. 3e) or visible light (Supplementary Fig. 4) treatment of Jurkat cells stimulated phosphorylation of ZAP-70 in a Lck-dependent manner. PLC-γ1 associates with the TCR/CD3 complex and is phosphorylated downstream of activation of the Src^20^,^22^,^23^. Indeed, we found that light induced phosphorylation of PLC-γ1 at tyrosine 783 (Fig. 3f). To confirm a functional requirement for Lck and PLC-γ1 we measured light-evoked Ca^2+^ responses in Lck- and PLC-γ1-deficient Jurkat T cells (Fig. 3g and h). Light-induced Ca^2+^ increases were abolished in Lck-null cells and largely suppressed in PLC-γ1-deficient cells, a residual component may reflect expression of PLC-γ2 that can partly compensate for PLC-γ1^24^. In contrast, responses were fully restored in cells stably expressing PLC-γ1 (Fig. 3g and h). Further, genetic disruption of TCR expression attenuated the speed and magnitude of light-evoked Ca^2+^ signaling (Fig. 3g and i). These data indicate that light evoked Ca^2+^ responses in T cells require activation of Lck tyrosine kinase coupled to PLC-γ1 and an intact TCR complex facilitates this process. Finally, we examined the transport pathway underlying the light-evoked Ca^2+^ response. Figure 3j shows that the Ca^2+^ rise persisted in a Ca^2+^-free medium indicating the mobilization of Ca^2+^ from internal stores. In addition, the duration of the Ca^2+^ rise was significantly greater in Ca^2+^-containing medium, suggesting a delayed component due to entry of extracellular Ca^2+^. Taken together, these data support the hypothesis that light stimulates a Src kinase/PLC-dependent pathway in T cells to mobilize Ca^2+^ and activate Ca^2+^ entry.

### Light generates H_2_O_2_ in T cells

**Figure 4:**
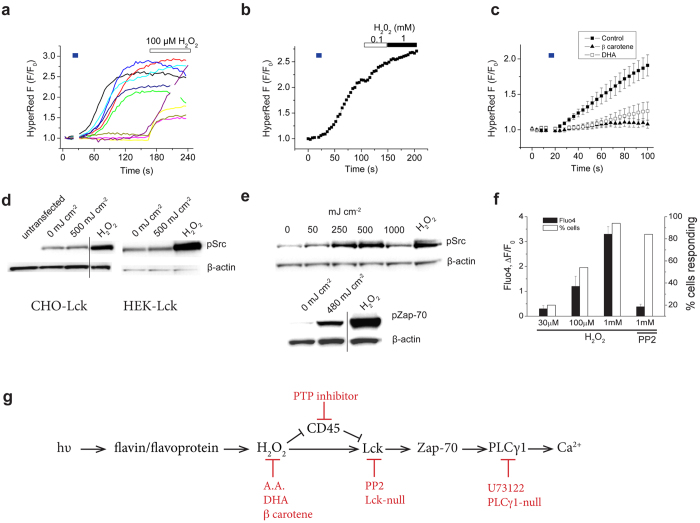
Blue light stimulates T cell production of H_2_0_2_. (**a** and **b**) Brief, blue light irradiation (30 mW cm^−2^) increases fluorescence of the genetically encoded H_2_0_2_ sensor, HyperRed, expressed in Jurkat cells. (**c**) Pretreatment with DHA (20 μM) or β carotene (100 μM) inhibited the light-evoked increase in HyperRed fluorescence (n = 10–15 cells per group). (**d**) H_2_0_2_ (2 mM, 2 min) but not blue light, increases phosphorylation of Lck expressed in CHO or HEK293 cells. (**e**) Blue light (10 mW cm^−2^, 600 mJ cm^−2^) and H_2_0_2_ (2 mM, 2 min) increase phospho-Lck and phospho-Zap-70 in Jurkat cells. Note that the immunoblots are cropped and the full blots are shown in Supplementary Fig. 9. (**f**) Mean changes in Fluo4 fluorescence and the% of responding Jurkat cells in response to 2 min treatment with H_2_0_2_ (30 μM to 1 mM) in the dark. F_0_ was measured using <10 mJ cm^−2^. (**g**) Proposed model for light-induced signaling in Jurkat cells; blue light photoreduces flavin that in the presence of 0_2_ generates H_2_0_2_, in turn, H_2_0_2_ activates Lck and downstream kinases. Red lines indicate key pharmacological or genetic inhibition steps used to support this pathway.

Mammals express two major classes of photoreceptors, opsins^3^ and cryptochrome flavoproteins (CRY1 & CRY2)^25^,^26^. These proteins utilize retinal or flavin/pterin-based chromophores respectively, to capture photons at discrete wavelengths. We screened Jurkat T cells for expression of the identified mammalian opsin genes^3^ and detected transcripts for Opsin 3 (*OPN3,* Supplementary Fig. 5). Further, we detected expression of both CRY1 & 2 in Jurkat cells (Supplementary Fig. 6). However, disrupting expression of OPN3 or CRY1 & 2 did not affect light-evoked Ca^2+^ changes (Supplementary Figs 5 and 6). The action spectrum for T cells (Fig. 2f), containing peaks in the near-UV and blue wavelengths, is characteristic of riboflavin or a flavin-binding protein^27^. UV and very high-irradiance blue light (1–5 W; 22–40 J cm^−2^) can activate cellular flavin-binding oxidases, leading to the formation of H_2_O_2_^28^ and therefore we explored for a similar mechanism in T cells. We found that the antioxidants, ascorbic acid and docosahexaenoic acid, blocked light-evoked Ca^2+^ responses (Fig. 3a) supporting a role for reactive oxygen species (ROS). However, light signaling persisted in the presence of the flavin oxidase inhibitor, diphenyliodonium, or mitochondrial electron transport chain inhibitors, antimycin and rotenone (Fig. 3a), arguing against a role for flavoprotein enzymatic activity or mitochondrial respiration. To directly and selectively monitor H_2_O_2_ levels we transfected Jurkat cells with the genetically-encoded H_2_O_2_ reporter, HyPerRed^29^. We found that a low dose of blue light (300 mJ cm^−2^, 30 mW) was sufficient to trigger H_2_O_2_ production in Jurkat cells (Fig. 4a). Subsequent stimulation of these light-sensitive cells with exogenous H_2_0_2_ (0.1–1 mM) produced little further increase in fluorescence, indicating that HyPerRed was almost completely oxidized by light-evoked signaling (Fig. 4a and b). Pretreating Jurkat cells with the anti-oxidants DHA and β-carotene (Fig. 4c) blocked the light-evoked increase in HyPerRed fluorescence. Flavins are well known to undergo photoreduction accompanied by production of several forms of reactive oxygen species^30^. Notably reduced flavins can react with O_2_ to generate H_2_O_2_^31^. To confirm this we measured production of H_2_0_2_ from irradiation of solutions containing riboflavin or flavin adenine dinucleotide (5 μM). In both cases, blue-light (600 mJ cm^−2^) produced H_2_0_2_ that was inhibited by DHA and β-carotene (Supplementary Fig. 7). Taken together, our data support a mechanism by which irradiation of flavin/flavoproteins in T cells generates H_2_0_2_.

H_2_O_2_ can activate Src kinases directly by covalent modification of cysteines^32^,^33^ or indirectly by inhibiting CD45, and thereby promoting phosphorylation of Src at the positive regulatory site (Lck Y394)^34^,^35^. To confirm a direct action we transfected CHO and Hek-293 cells with Lck and measured phospho-Lck levels in response to blue light (500 mJ cm^−2^) or exogenous H_2_O_2_ (2 mM, 2 min). In both cell lines light failed to activate Lck, whereas H_2_O_2_ produced a robust increase in levels of phospho-Lck (Fig. 4d). In contrast, in Jurkat cells, both blue light and H_2_O_2_ strongly activated Lck and Zap-70 (Fig. 4e). Further, exogenous H_2_O_2_ (30–1000 μM, 2 min) increased intracellular [Ca^2+^] in Jurkat cells, predominantly by increasing the number of responsive cells (Fig. 4f, Supplementary Fig. 8), and this effect was inhibited by PP2. These data show that H_2_O_2_ is sufficient to activate Lck and provide a causal nexus between blue light and activation of Ca^2+^ signaling. Figure 4g illustrates a putative signaling pathway with key pharmacologic and genetic interventions. In this model H_2_O_2_, generated by flavin photo-irradiation, activates Lck either directly and/or indirectly via CD45 to trigger signaling via Zap-70 and PLC-γ1. The precise role of CD45 is unclear since pharmacologic block of CD45 *per se* would also affect downstream Lck function by affecting the positive and/or negative regulatory sites Y394 and Y505 respectively^34^,^35^.

### Light enhances T-cell motility

**Figure 5:**
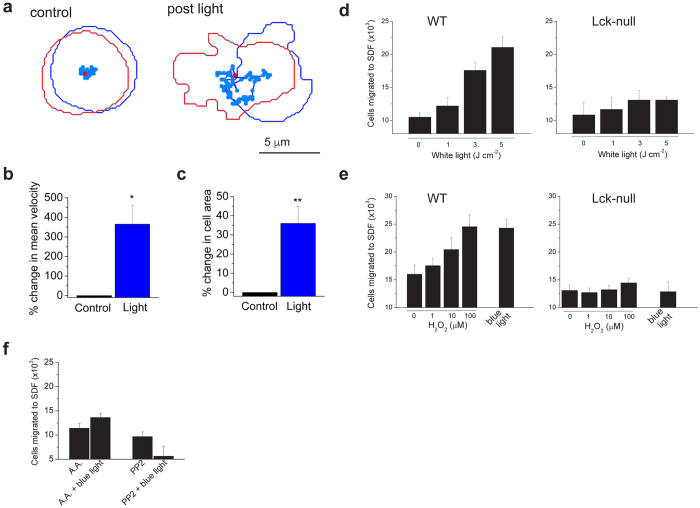
Light stimulates T-cell motility. (**a**) Blue-light enhanced random motility in a mouse CD8+ T cell. Plot of cell shape and trajectory for 4 minutes before and after irradiation (100 s, 120 mJ cm^−2^). The first and last cell images/positions are shown as blue and red respectively. (**b** and **c**) Blue light (120 mJ cm^−2^) increased T cell mean velocity and peak surface area (n = 5, *P < 0.05, **P < 0.01). (**d** and **e**) Light or exogenous H_2_0_2_ increased chemotaxis (2 hr) of Jurkat T cells, but not Lck-null cells, to SDF-1α. Note that cells were irradiated in (**d**) with broad spectrum or in (**e**) with blue light (600 mJ cm^−2^). Data are the mean of 3–4 experiments. (**f**) Abscorbic acid (A.A., 10 mM) and PP2 (2 μM) inhibit blue-light stimulation of chemotaxis in Jurkat cells (n = 3–4).

Next, we considered how photosensitivity might affect T-cell function. We observed that blue-light irradiation altered the morphology of T cells, inducing the extension of lamellipodia (Fig. 1a and Supplementary Movies 2 and 3). Since motile lamellipodia are associated with cell migration^36^ we measured effects of light on T-cell motility by tracking the movement of activated CD8+ T cells across a fibronectin-coated surface. Figure 5a–c shows that low levels of blue light (120 mJ cm^−2^) increased random T-cell motility and the peak surface area (reflecting spreading lamellipodia), an effect that persisted following irradiation. Thus, T cells exhibit photokinetic behavior. Next, we measured effects of light on chemotaxis. Figure 5d shows that irradiation with white light (1–5 J cm^−2^) enhanced migration of Jurkat T cells towards the T-cell chemokine, stromal-derived factor. Light increased chemotaxis in a dose-dependent manner with a peak 100% increase observed at irradiances greater than 2 J cm^−2^. In contrast, the same doses of light failed to stimulate chemotaxis in Lck-null Jurkat cells (Fig. 5d) confirming a requirement for Src kinase signaling. Next, we asked whether exogenous H_2_0_2_ could mimic the effects of light. Figure 5e shows that H_2_0_2_ dose-dependently increased chemotaxis in Jurkat cells with 100 μM producing an effect equivalent to that of blue-light irradiation. Again, Lck-null cells were resistant to the actions of H_2_0_2_ and light (Fig. 5e). Finally, we found that treating wild-type Jurkat cells with abscorbic acid or PP2 blocked the stimulatory effects of blue light (600 mJ cm^−2^, Fig. 5f). Taken together, these data show that H_2_0_2_, acting via Lck, is both necessary and sufficient for light-evoked stimulation of T cell motility.

## Discussion

Our data show that T cells possess the intrinsic capacity to sense and respond to light. We show that blue light triggers the production of H_2_O_2_ in T cells *in vitro*. In turn, light-evoked H_2_O_2_ leads to activation of Src, Zap-70, PLC-γ1 and intracellular [Ca^2+^] (see Fig. 4g) and these effects are accompanied by alterations in T cell motility. Importantly, these effects of light were reproduced by exogenous H_2_O_2_ and occurred in a Lck-dependent manner. Our experiments exclude photosensitization of dyes employed in some measurements. First, activation of Src and Zap-70 measured biochemically, as well as T cell migration occurred at similar light doses that produced Ca^2+^ responses. Second, the spectral sensitivity of T cells with peaks at both ~350 nm (UV) and ~470 nm (blue) does not match the absorption profile for Fluo4. Rather, this spectrum is consistent with involvement of flavin/flavoproteins. Flavins are abundant in mammalian cells with concentrations approaching 50 μM measured in cardiac myocytes^37^, albeit much of this flavin is protein bound. Flavins are readily photoreduced leading to the generation of ROS including H_2_O_2_^30^,^31^. Accordingly, we confirmed that blue light irradiation of flavin-containing solutions, at similar doses (600 mJ cm^−2^) used on T cells, produced H_2_O_2_ *in vitro*. There is also evidence in mammalian cells for high energy UV and blue light activating flavin oxidases and generating ROS^28^. Further, high frequency UV light (<280 nm) activates a mammalian “UV response” involving Src kinase and the *c-jun* and *c*-*fos* genes^38^, and this effect is blocked by the antioxidant N-acetyl cysteine. These light-evoked ROS signals are reported to emanate from peroxisomal^28^ or mitochondrial compartments^39^. It should be pointed out that sensitivity of T cells to light that we report here using low light (4–30 mW cm^−2^, 50–600 mJ cm^−2^) is much greater than these earlier reports employing very high irradiances and doses (1–3 W cm^−2^, 20–40 J cm^−2^). Further, light signaling in T cells persisted in the presence of flavin oxidase or mitochondrial electron transport chain inhibitors ruling out enhanced flavin enzymatic activity or mitochondrial respiration as a source of H_2_O_2_.

Interestingly, we found that photosensitivity was greater in activated compared to naïve T cells. This might reflect clustering of TCR complexes in lipid micro domains that occurs in activated cells^40^. The TCR provides a scaffold for kinases and adaptor proteins including Src, Zap-70 and PLC-γ1^20^,^41^, all of which we found are activated by blue light. Indeed, inhibiting TCR expression greatly attenuated light-evoked Ca^2+^ responses. Further, upon T cell activation flavoprotein rich mitochondria translocate toward the immune synapse^42^, potentially providing a greater source for light-evoked ROS. Finally, activation of T cells alters their antioxidant capacity; the membrane bound ROS scavenger, peroxiredoxin 1, is inhibited following TCR stimulation to permit microdomains of elevated ROS^43^, and this may augment light-induced H_2_O_2_. This H_2_O_2_ can activate Src kinases either directly^32^,^33^ as we confirmed in cell lines, and/or indirectly, by inhibiting activity of the protein phosphatase CD45^34^, leading to enhanced tyrosine phosphorylation at the Src positive regulatory site, Y394. Interestingly, higher light doses attenuated the activation of Src and Zap-70 kinases and this may partly reflect ROS-mediated inactivation of Src^44^.

Importantly, we show that light potently affects T cell motility, stimulating random cell movement and chemotaxis. The effects on light on motility were maintained after irradiation and, since phototaxis requires a specialized apparatus to detect the directionality of light^45^, can be most parsimoniously explained as a photokinetic effect. Photokinesis is common in many eukaryotic microorganisms^45^, but to the best of our knowledge this constitutes the first report of photokinesis in a mammalian cell. We found that light-evoked chemotaxis required Lck (interrogated both pharmacologically and genetically) and was replicated by exogenous H_2_O_2_. ROS signaling is known to affect motility in various cells^46^ although a role for ROS in T cell motility has not been previously established. Src kinases are also recognized regulators of leukocyte motility^47^, activating phosphoinositide 3 (PI3)-kinase γ and Rho GTPases, that in turn affect cell polarization, membrane protrusions, and motility. Thus, a light evoked H_2_O_2_/Src signaling pathway appears to underlie T cell photokinesis.

The identification of T cell photosensitivity has implications for both immunological research and immunobiology. In the experimental setting, researchers exploiting light-based approaches, optogenetics for example, should consider the intrinsic actions of light on T cells. In the biological setting sunlight, particularly in the visible range, may impact the function of skin T cells. Although it needs to be confirmed *in vivo*, we propose that light may enhance the motility and migration of T cells through skin. Skin T cells comprise a population of memory cells performing immune surveillance (that can be activated in the skin by antigen-presenting cells) and others cell actively recruited by inflammation^19^. These cells may be exposed to considerable solar radiation. Bright sunlight has a peak irradiance of 120 mW cm^−2^ and approximately 40% of the energy is contained in the visible spectrum with a peak flux in the blue-green region (450–500 nm)^6^. Blue light can penetrate several millimeters through skin. Indeed, blue-light irradiation of mouse skin has been used to drive subcutaneous optogenetic implants^48^. Further, our observation that T cell photosensing involves an integration of the light signal (photon counting) indicates that photosignaling can proceed even with weak light levels given sufficient time.

In summary, our data show that T cells possess intrinsic sensitivity to blue light and that light operating via a H_2_O_2_ signaling pathway enhances T-cell motility.

## Materials and Methods

All experimental procedures involving mice were approved by the Georgetown University Animal Care and Use Committee and conformed to National Institutes of Health guidelines.

### Cell Culture

Jurkat, J.RT3-T3.5 (T cell receptor β chain-deficient), J. Cam1.6 (Lck kinase deficient), J. gamma1 (PLC-γ1-null), J. gamma1WT (PLC-γ1 rescue) and D1.1 cell line cells (American Tissue Culture Collection), were cultured in RPMI medium 1640 (HyClone) supplemented with 2 mM L-glutamine, 50 μM 2-ME, 1 mM sodium pyruvate, 10 mM HEPES, 1% penicillin and streptomycin, and 10% FBS (HyClone). Cells were maintained at density 0.5–1 × 10^6^/ml. Mouse (C57/Bl6, Jackson Labs) T cells were purified from splenocytes or lymph nodes using MagCellect Mouse CD3 T Cell Isolation kit (R&D Systems). Human lymphocytes were separated from whole blood (obtained from Research Blood Components, Brighton, MA, USA) with Lymphoprep (Axis-Shield) density gradient centrifugation and purified using Dynabeads Untouched Human T cell Kit (Invitrogen). Cells were cultured for 48 h in RPMI-1640 medium supplemented with Il-2 10 ng/ml (Miltenyi Biotec) and ConA 5 μg/ml (Sigma Aldrich). Il-2 and Con A were then removed for 18 h–48 h prior to experiments. In some experiments mouse cells were activated with plate bound anti-CD3 antibody (10 μg/ml, 145-2C11; Biolegend) and anti-CD28 (0.5 μg/ml, 37.51; Biolegend) for 48 h, and rested overnight prior to experiments. Naive CD3+ T cells were isolated from mouse lymph nodes cultured for 12–48 hrs with a low concentration of IL-2 (2 ng/ml).

### Irradiation and Ca^2+^ imaging

Cells were plated on glass coverslips in serum depleted (0.5% FBS) buffer in the dark for 90–120 minutes prior to imaging. The buffer contained in mM: 140 NaCl, 4 KCl, 10 HEPES, 10 glucose, 1.2 CaCl_2_, 1 MgCl_2_ pH 7.3. Under these conditions cells loosely adhered to the coverslip. Cells were irradiated with an Argon laser (488 nm) on a Zeiss confocal microscope or via the epifluorescent port of a Nikon TE2000 microscope using the following excitation filters (nm): 355 ± 25, 370 ± 5, 400 ± 20, 440 ± 5, 480 ± 15, 510 ± 5 and 535 ± 25. DIC/brightfield images were obtained at 561 nm (Zeiss microscope) or using low intensity white light irradiance (tungsten-halogen lamp) that evoked minimal activation of Ca^2+^. For white-light treatment we used a full spectrum irradiance lamp (400–700 nm, Full Spectrum Solutions, Jackson, MI). Visible sunlight energy was calculated assuming ~40% energy (of total 120 mW cm^−2^) in the 400–700 nm range. Irradiance was measured with a X-Cite Optical Power Meter calibrated for each wavelength, coupled to an objective plane power sensor (Lumen Dynamics Group Inc. Mississauga, Ontario). Temperature, unless otherwise indicated, was 22–23 °C.

For Ca^2+^ imaging, cells were loaded with 5 μM Fluo4-AM or Rhod-2-AM for 40 min and were washed in serum-depleted (<0.5% FBS) buffer. Fluo4 was excited at 480 ± 15 nm (0.5–30 mW cm^−2^) and emitted fluorescence was passed through a 535 ± 25 bandpass filter, Rhod-2 was excited at 540.5 ± 12.5 nm and emitted fluorescence measured at 630 ± 30 nm. Fluorescence was captured by a SPOT RT digital camera (Diagnostic Instruments, Sterling Heights, MI) and read into a computer. Analysis was performed offline using Simple PCI software (Compix Inc, Sewickley, PA). Maximal Fluo4 fluorescence (*F*_max_) was obtained by application of 10 μM ionomycin at the end of the experiment as described previously^49^. For analysis of cumulative cell activation (% activation) the half-maximal Ca^2+^ increase for each cell was measured and scored in 2–4 s bins. A Ca^2+^ response was defined as greater than 20% change in fluorescence from the baseline.

### HyPerRed imaging

For imaging H_2_O_2_, Jurkat cells were transfected by electroporation with 2 μg HyperRed cDNA (Addgene). Cells were returned to culture for 48 h prior to measurements. HyperRed was excited with a 540.5 ± 12.5 nm bandpass filter and emitted fluorescence measured at 620 ± 30 nm.

### Pharmacology

Jurkat cells were pretreated with the following compounds: U73122 (500 nM, 5 min) U733433 (5 μM, 5 min), wortmanin (1 μM, 40 min), genestein (100 μM, 40 min), PP2 (3 μM, 40 min), PP3 (3 μM, 40 min), CD45 inhibitor (N-(9,10-Dioxo-9,10-dihydro-phenanthren-2-yl)-2,2-dimethyl-propionamide; 1 μM, 120 min), ascorbic acid (10 mM, 40 min), docosahexaenoic acid, DHA (5–20 μM, 20 min), ββ carotene (100 μM, 20 min), apocynin (100 μM, 40 min), antimycin/rotenone/oligomcyin (20:20:5 μM, 20 mins) and diphenyliodonium (50 μM, 40 mins). All treatment groups were compared to control cells incubated for the same period in normal buffer.

### Chemotaxis and cell motility assays

Chemotaxis was assayed using the CytoSelect 96-well Cell Migration Assay Kit (3 μm pore, Cell Biolabs, INC.) 5 × 10^5^ Jurkat cells were suspended in serum free RPMI media and loaded into the upper chamber of the transwell. The bottom chamber contained media and 40 ng/ml SDF-1α (Peprotech Inc., Rocky Hill, NJ). Cells were irradiated with visible light (5–10 mW cm^−2^) or a blue light photodiode array (470 nm, 10 mW cm^−2^) and then incubated for 120 min at 37 °C in a CO_2_ incubator. Migrated cells were counted manually using a hemocytometer. Cell motility assays were performed on fibronectin (2 μg/ml) coated coverslips at 30–32 °C. Individual cells were tracked using *CellTrack* software^50^. We analyzed cells for 4 min before and after blue-light irradiation.

### Proliferation assays

Splenocytes were seeded in 96-well plates (2 × 10^5^ cells per well) pre-coated with 1–10 μg/ml anti-CD3e (clone 145-2C11, BD Pharmingen). After 72 hours, cells were counted using MTT assay.

### Immunoblotting

Jurkat cells were serum starved for 18 h in RPMI-1640 medium containing 0.5% BSA. 2 × 10^6^ cells were suspended in HANKS buffer containing 0.5% BSA and irradiated. Cells were lysed in RIPA buffer containing 50 mM Tris-HCl, pH 7.4, 1% NP-40, 0.25% Na-deoxycholate, 150 mM NaCl, 1 mM EDTA, 1 mM PMSF, 1 mM Na_3_VO_4_, 1 mM NaF, and complete protease inhibitor mixture (Sigma Aldrich), followed by centrifugation at 14,000 rpm at 4 °C for 10 min. After protein concentrations were quantified, cell extract was mixed with NuPAGE® *LDS* Sample Buffer and samples were loaded onto 4–12% polyacrylamide gels and proteins were resolved by SDS-PAGE in a NuPAGE electrophoresis system (Invitrogen). Proteins were transferred to Polyvinylidene Difluoride (PVDF) membrane. Membranes were blocked for 1 h at room temperature with 5% skim milk and probed once with specific antibodies overnight at 4 °C. Immunoblots were then incubated with horseradish peroxidase-conjugated secondary antibodies diluted 1:5,000 for 1 h at RT. Protein bands were visualized by the enhanced chemiluminescence reaction method (SuperSignal West Pico Chemiluminescent Substrate Pierce). Band densities were quantified using ImageJ. We used the following antibodies: Rabbit Phospho-Zap-70 (Tyr319) (Cell Signaling Technology), Rabbit Phospho-Src Family (Tyr416) (Cell Signaling Technology), Mouse β-Actin, OPN3, CRY1, CRY2, PLCγ-1 (Santa Cruz Biotechnology, Inc), PLCγ-1-phospho (Y783) (BioSource).

### RT-PCR

We measured mRNA expression of the following opsins by RT-PCR: OPN1 (long and short-wave), OPN2, OPN3, OPN4, OPN5, peropsin and RGR using published primers.

### RNA interference

Jurkat cells were transfected using the Amaxa nucleofection technology. Cells were resuspended in Amaxa Cell Line V nucleofector solution (2 × 10^6^ per reaction) according to the manufacturer’s instructions. 5 μM of siRNA (Ambion) were added and nucleofected using manufacturer program C16. Cells were then transferred to supplemented RPMI-1640 medium and the gene knockdown was analyzed after 24, 48 and 72 hours at mRNA and protein level.

### Chemicals

U73122, U733433 and CD45 inhibitor (CAS 345630-40-2) were purchased from Calbiochem. PP2 and PP3 were from Tocris. All other chemicals were from Sigma-Aldrich.

### Statistical analysis

Data are presented as means ± SE. Significance was assessed with the Student’s *t* test (*P* < 0.05 for significance).

## Additional Information

**How to cite this article**: Phan, T. X. *et al*. Intrinsic Photosensitivity Enhances Motility of T Lymphocytes. *Sci. Rep.* **6**, 39479; doi: 10.1038/srep39479 (2016).

**Publisher's note:** Springer Nature remains neutral with regard to jurisdictional claims in published maps and institutional affiliations.

## Supplementary Material

Supplementary Information

Supplementary Movie 1

Supplementary Movie 2

Supplementary Movie 3
