# Supplementary material for: Intrinsic Photosensitivity Enhances Motility of T Lymphocytes
Source: Sci Rep. 2016 Dec 20;6:39479. doi: 10.1038/srep39479 (PMC5171715; doi:10.1038/srep39479)
Supplement: Supplementary Information [file srep39479-s1.pdf]

Supplementary Information

"Intrinsic Photosensitivity Enhances Motility of T Lymphocytes" by Phan X. Thieu, Barbara Jaruga, Sandeep C. Pingle, Bidhan C. Bandyopadhyay, & Gerard P. Ahern

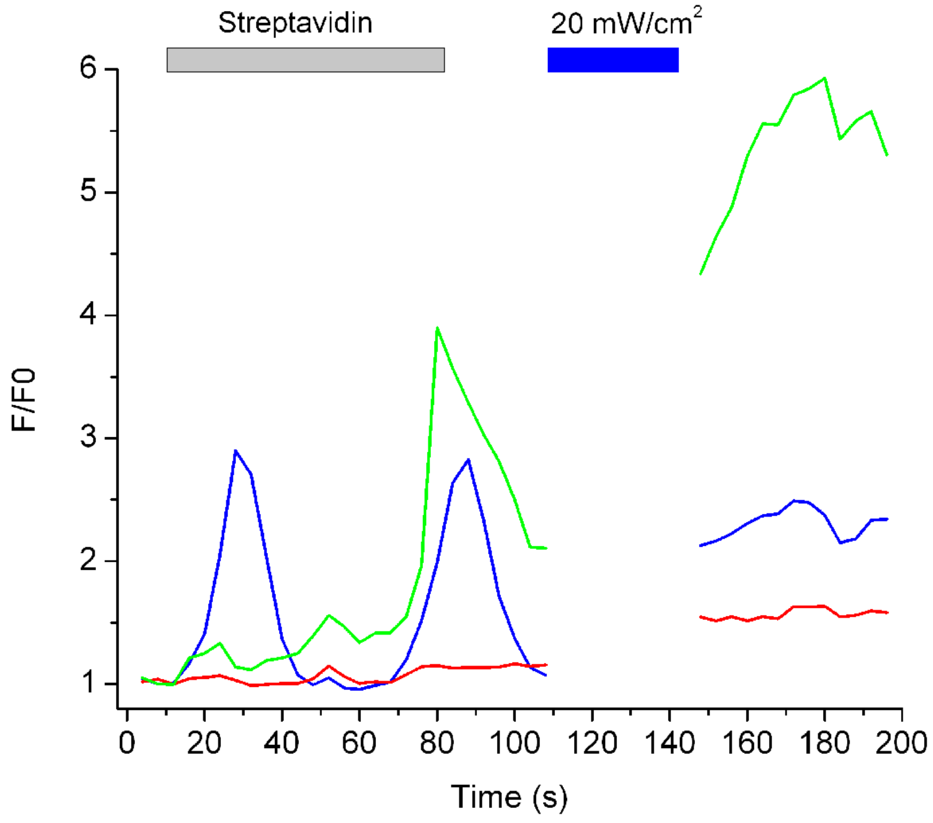

Supplementary Figure 1. Comparison of Ca^{2+} responses evoked by T cell receptor mediated signaling and blue light irradiation. Mouse CD4+ T cells were incubated with 5 µg/ml biotinylated anti-CD3 antibody (BD Biosciences) for 10 min at RT followed by cross-linking with 5 µg/ml streptavidin (Pierce Chemical Co.) as indicated. A 30 s pulse of blue light (20 mW cm^{-2}) was used to trigger photosignaling. Ca^{2+} imaging was performed in Fluo4 loaded cells. The dye was excited with low intensity blue light (0.8 mW cm^{-2})

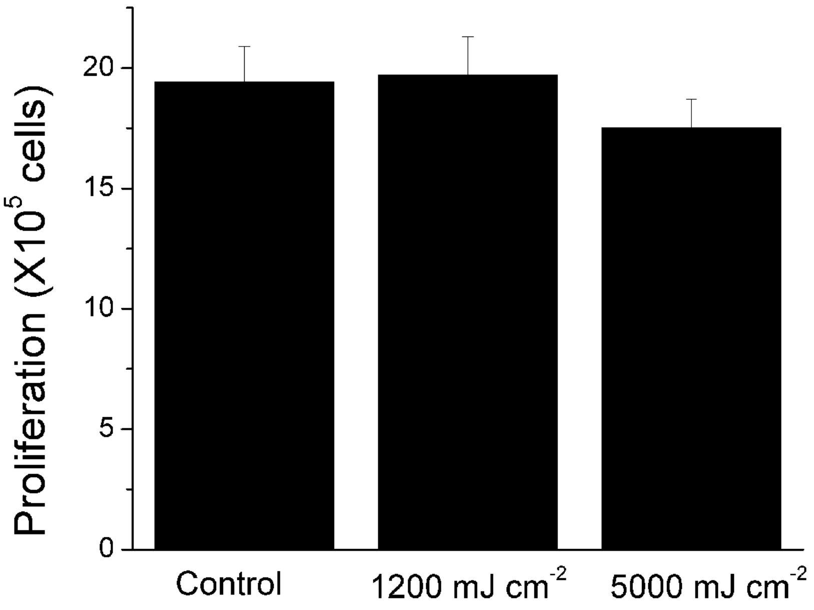

Supplementary Figure 2. Light treatment does not affect T cell proliferation. Mouse splenocytes were stimulated with plate bound anti-CD3 antibody (10 μg/ml) for 3 days with or without daily irradiation with full spectrum light. Cell were counted using MTT assay.

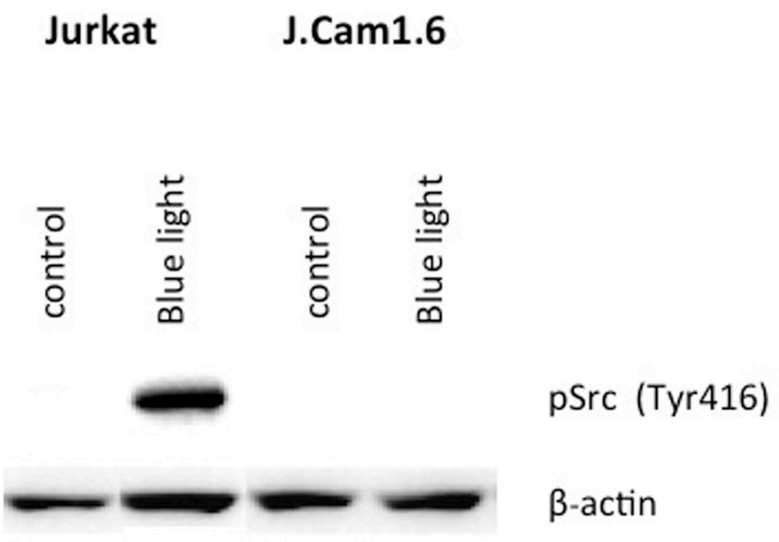

Supplementary Figure 3. Lck activation by light in Jurkat and Lck-null Jurkat cells. Blue light induces phosphorylation of Src kinase as detected with a generic pSrc antibody (mouse Y416). The prominent immunoreactive band is absent in J.Cam1.6 (Lck-null) cells confirming predominant activation of Lck.

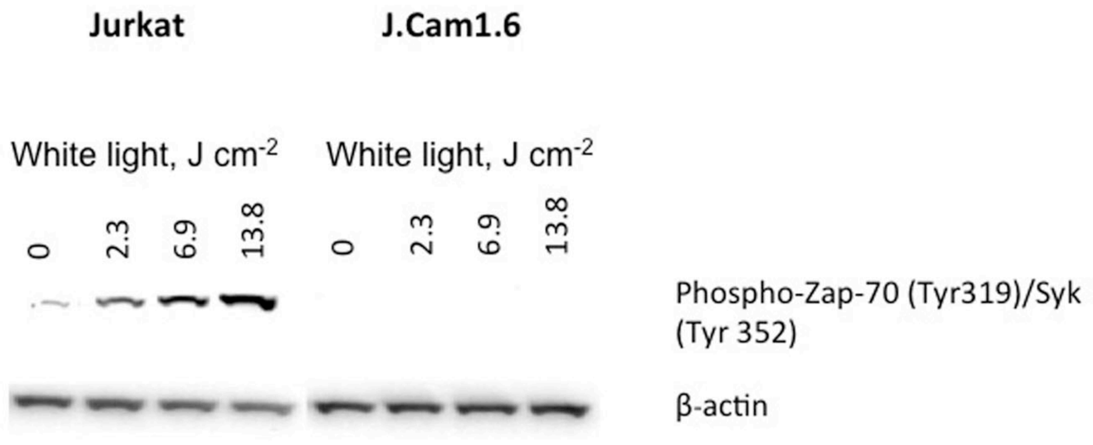

Supplementary Figure 4. White light activates Zap-70 in a Lck-dependent manner. Induction of phospho-Zap-70 by white light in Jurkat and J.Cam1.6 (Lck-deficient) cells.

A

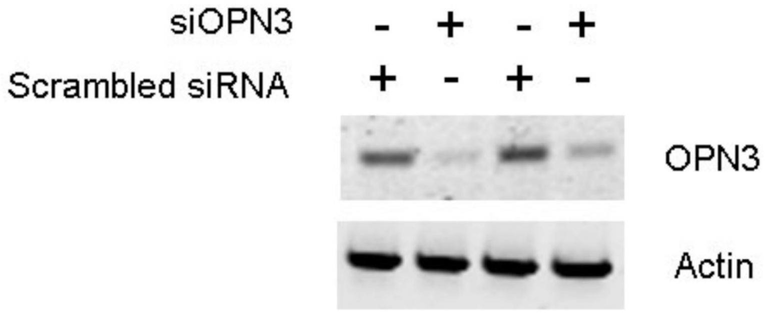

B

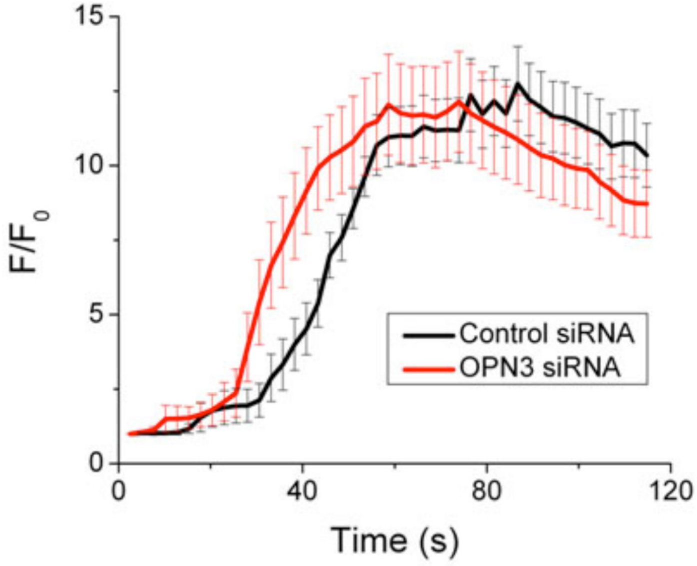

Supplementary Figure 5. Opsin 3 (OPN3) does not contribute to light signaling in Jurkat cells. **A**, inhibition of Opsin 3 mRNA expression in Jurkat cells by siRNA. **B**, inhibition of Opsin 3 mRNA expression does not disrupt blue light-evoked Ca^{2+} signaling.

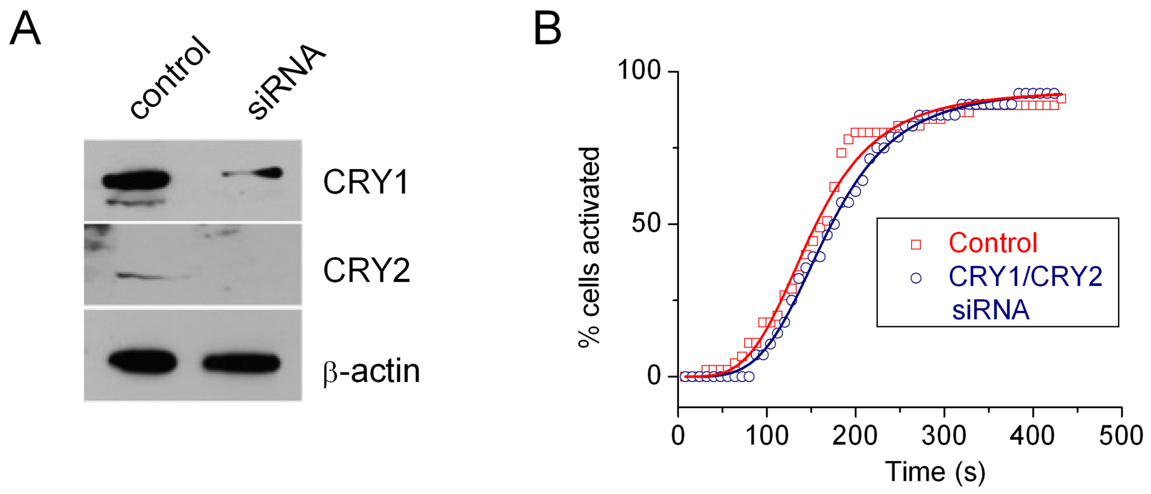

Supplementary Figure 6. Light activates Ca^{2+} independently of Cryptochrome proteins. Inhibiting protein expression of cryptochrome 1&2 (CRY1&2) (A) in Jurkat cells does not affect light-evoked Ca^{2+} signaling (B).

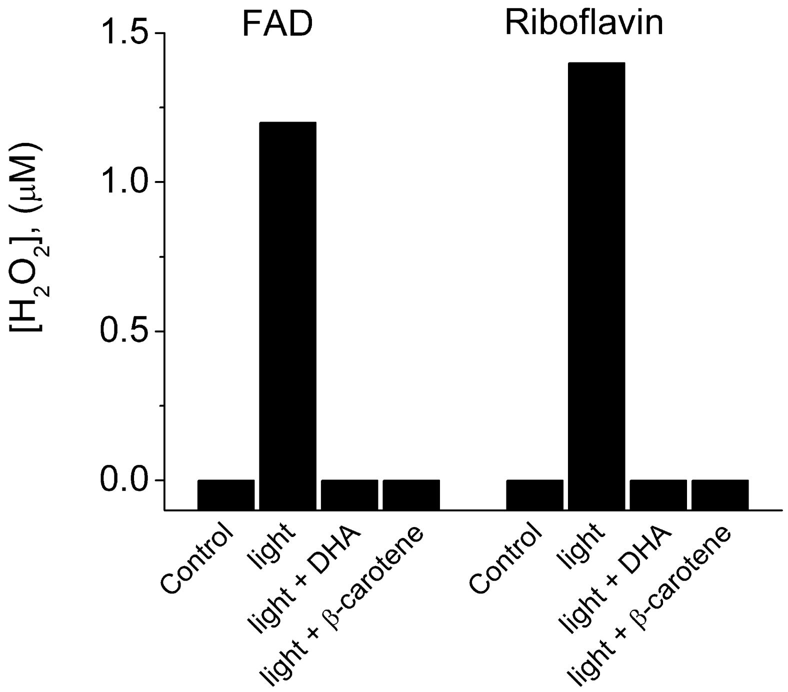

Supplementary Figure 7. Blue light irradiation of riboflavin and flavin adenine dinucleotide generates H_20_2. Solutions containing 5 μM of riboflavin or flavin adenine dinucleotide were irradiated with 600 mJ cm^{-2} blue light with or without docosahexoenoic acid or β-carotene (10 μM). [H_20_2] was measured using a colorimetric Oxired assay (Abcam).

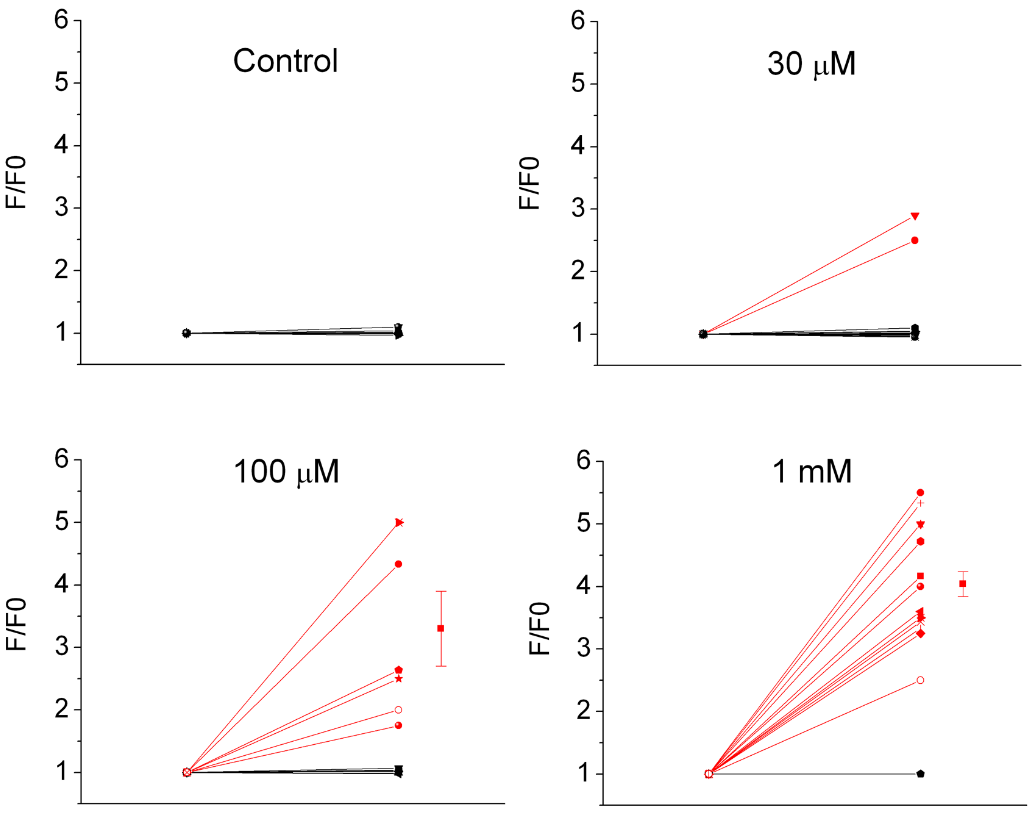

Supplementary Figure 8. H_20_2 increases intracellular [Ca^{2+}] in Jurkat cells. Fluo4 fluorescence in individual cells before and after a two-minute exposure to 0, 30, 100 μM and 1 mM H_20_2 (n=12-15 cells). Non-responding and responding cells are denoted as black and red respectively. Note that H_20_2 in a concentration dependent manner increases the number of responding cells. Mean F/F_o (3.3 ± 0.6 and 4.1 ±0.2) was not significantly different (P=0.15) for 100 μM and 1 mM treatments. F_0 was measured using <10 mJ cm^{-2} blue light that did not affect subsequent fluorescence measured 2 minutes later.

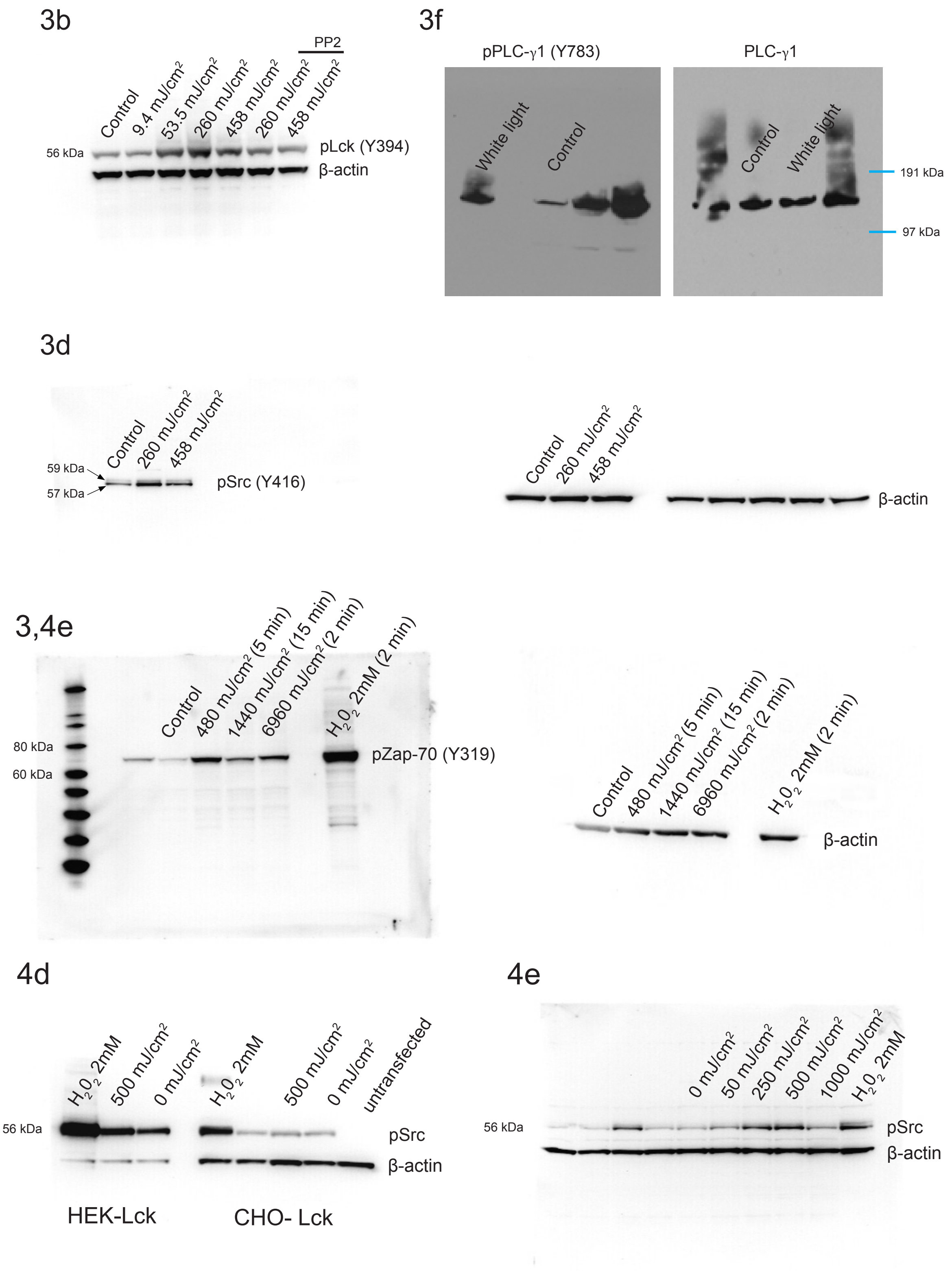

Supplementary Figure 9. Full-sized immunoblots for Figures 3b-f, 4d&4e.

Supplementary Movie 1. Blue light irradiation (5 mW cm^{-2}) stimulates an increase in [Ca^{2+}] in Jurkat T cells; 7.5x speed.

Supplementary Movie 2. Single cell Ca^{2+} and motility response to blue light (488 nm laser 2.7 mW cm^{-2}; 20x speed).

Supplementary Movie 3. Blue light triggers extension of lamellipodia in Jurkat cells (488 nm laser 2.7 mW cm^{-2}, 37ºC, 20x speed).
